# The Frailty Index, Missing Data, and Imputation

**DOI:** 10.1093/geroni/igaa057.3389

**Published:** 2020-12-16

**Authors:** Glen Pridham, Andrew Rutenberg

**Affiliations:** Dalhousie University, Halifax, Nova Scotia, Canada

## Abstract

The frailty index (FI) is a summary measure of health during aging that is defined by the average number of ‘things wrong’, i.e. health deficits, across a sundry of lab, clinical, and questionnaire measurements. Missing data are ubiquitous in aging studies. Although the FI appears to have robust predictive power—even when ignoring missing data, there has not been a systematic study of the consequences of imputation when used in the principle investigation. We investigated the standard imputation methodology, multiple imputation using chained equations (MICE), and other missing data methods, in terms of prediction of mortality and statistical power using the 2003/04 and 2005/06 NHANES datasets. When we masked known data completely at random, we observed that available case analysis incorrectly estimated the true variance of the FI leading to potential problems in hypothesis testing, whereas imputation helped mitigate this effect. We also observed that the default imputation methods from MICE showed a significant increase in FI relative to the ground truth together with a decrease in predictive power, hence we suggest other options when performing imputation with NHANES. The underlying missing mechanism in NHANES is not random and appears to be important, for example survival curve analysis showed that the top half of patients with the most missing data died significantly younger than the bottom half.

